# Pronounced Inflammatory Response to Endotoxaemia during Nighttime: A Randomised Cross-Over Trial

**DOI:** 10.1371/journal.pone.0087413

**Published:** 2014-01-27

**Authors:** Mahdi Alamili, Klaus Bendtzen, Jens Lykkesfeldt, Jacob Rosenberg, Ismail Gögenur

**Affiliations:** 1 Department of Surgical Gastroenterology, University of Copenhagen, Herlev Hospital, Herlev, Denmark; 2 Institute for Inflammation Research, Department of Rheumatology, Copenhagen University Hospital, Rigshospitalet, Denmark; 3 Department of Veterinary Disease Biology, Faculty of Health and Medical Sciences, University of Copenhagen, Copenhagen, Denmark; University of Leuven, Rega Institute, Belgium

## Abstract

**Background:**

Circadian variation in bodily functions has been shown to impact health in acute and chronic medical conditions. Little is known about the relationship between circadian rhythm and sepsis in humans. We aimed to investigate circadian variations in the host response in a human endotoxaemia model.

**Design and Methods:**

A cross-over study, where 12 healthy young men received *E. coli* endotoxin (lipopolysaccharide, LPS) 0.3 ng/kg at 12 noon and, on another day, at 12 midnight. Blood samples were analysed for pro- and anti-inflammatory cytokines: tumour-necrosis factor (TNF)-alpha, soluble TNF receptors (sTNF-R)-1 and -2, interleukin (IL)-1beta, IL-1 receptor antagonist (IL-1Ra), IL-6, and IL-10 as well as YKL-40 and the oxidative stress markers malondialdehyde (MDA), ascorbic acid (AA) and dehydroascorbic acid (DHA) before and at 2, 4, 6 and 8 hours after LPS administration.

**Results:**

The levels of MDA and IL-10 where significantly higher during the day time (*P*<0.05) whereas levels of TNF-alpha, sTNF-RI, sTNF-RII, IL-1Ra, IL-6, and YKL-40 were higher (P<0.01 for all comparisons) during the night time. No significant differences were seen in the levels of AA and DHA.

**Conclusion:**

A day-night difference in the acute phase response to endotoxaemia exists in healthy volunteers with a more pronounced inflammatory response during the night time. This circadian difference in the response to endotoxaemia may play an important role in the clinical setting and should be investigated further.

## Introduction

The existence of circadian variation in acute medical settings is well established [1]–[3]. The occurrence of acute myocardial infarction, sudden cardiac death, pulmonary thromboembolisms and stroke exhibits a distinct circadian variation [4]. In surgery, both major and minor abdominal surgery results in circadian disturbances [5] including a circadian distribution of postoperative cardiovascular events [4].

Although circadian variations exist in the circulating levels of lymphocytes, macrophages and neutrophils, and cytokines released from these cells [6], [7], little is known about the circadian variations in the immunoinflammatory response accompanying septic conditions, where cytokines and oxidative stress play a crucial role [8]–[11]. Up to 4% of patients undergoing surgery develop postoperative sepsis, and 70% of these develop severe sepsis [12]. The mortality for patients with sepsis is high, ranging from 16% to 46% for patients with septic shock [13].

The human endotoxaemia model is an experimental systemic inflammatory model that is induced by the intravenous administration of lipopolysaccharide (LPS) endotoxin, mainly from Gram-negative bacteria [14]. It is a suitable model for investigating sepsis under controlled conditions. Endotoxaemia results in an increase in the plasma levels of pro-inflammatory mediators/marker (YKL-40, TNF-α, IL-1β and IL-6) and anti-inflammatory mediators (IL-1Ra, IL-10, sTNF-RI and sTNF-RII) [14], [15]. The endotoxaemia generates free radicals (e.g. reactive oxygen species) that induce an oxidative stress response, resulting in cell and tissue damage, called oxidative damage. The body counteracts the oxidative stress by antioxidants and antioxidative enzymes [16], [17].

We aimed to investigate, in a randomised cross-over trial in healthy subjects, the circadian variation in the inflammatory and oxidative stress response after experimental endotoxaemia.

## Methods

### Ethical statements

Approval from the Regional Committee on Biomedical Research Ethics (H-2-2010-010), The Danish Data Protection Agency, and the Danish Medicine Agency (EudraCT-no. 2009-017360-1) were obtained, and all study subjects gave written informed consent before enrolment in the study. The Good Clinical Practice (GCP) Unit at Copenhagen University monitored the study. The trial was registered at www.clinicaltrials.gov (NCT 01087359).

### Design and procedure

The study was a randomised, open cross-over trial including healthy subjects between ages 18 and 40. The trial consisted of two study days. On the first day, 0.3 ng/kg of *E. coli* LPS (Lot G3E069; US Pharmacopeia Convention, Rockville, MD, USA) was administered at 12 noon. In the second study, the same dose was given at 12 midnight. The participants were excluded if one of these criteria were present: Any use of tobacco, alcohol abuse, any medication, or overt infection. Individuals were not included if they suffered from chronic diseases, allergy against LPS endotoxin or insomnia, or if they had had any infections in the past 14 days before study initiation. Before inclusion, the subjects underwent a clinical examination to confirm that there were no unrecognized diseases. In the week before each study day, the participants were instructed not to take caffeine or alcohol and to sleep in a standardized manner (8 hours) between 11 p.m. and 8 a.m. A priori wash-out period between the two study days was 25 days and if the subjects had any sort of infection the wash out period was extended with at least two weeks from the last day of the infection. On each study day, intravenous catheters were inserted in both cubital veins. LPS endotoxin was administered as intravenous bolus injections. Blood samples were obtained at baseline, i.e. before the onset of endotoxaemia, and 2, 4, 6 and 8 hours after LPS injection. All subjects were monitored hourly during the endotoxaemic phase with measurements of blood pressure, temperature, and heart rate. Blood pressure and heart rate were measured using Micro life BP A100 Plus (Microlife, Widnau, Switzerland), and temperature was measured by tympanic thermometer Genius 2 (Covidien Ilc, Boulder, CO, USA).

All subjects had a private room. During night time endotoxaemia the subjects were assigned to follow the same sleep pattern as kept before each study day, i.e. 8 hours of sleep between 11 p.m. and 8 a.m. Subjects slept in complete darkness. Blood samples and vital parameters during night were taken with a pen light directed away from the patient eyes to minimize both disturbance and light exposure. The subjects were prohibited to drink any caffeine-containing beverages (coffee, tea) and alcohol on both experimental days.

### Laboratory analyses

#### Inflammatory markers

Blood was drawn into tubes containing EDTA and centrifuged at 3,100 G for 3 minutes and then stored at −80° C until analysis. The cytokines tumour necrosis factor alpha (TNF-α), interleukin-1β (IL-1β), interleukin-1Ra (IL-1Ra), interleukin-6 (IL-6), interleukin-10 (IL-10), and the soluble TNF receptors (sTNF-R) I and -II were measured in a Luminex 100 IS analyser (Luminex Corporation, Austin, TX, USA) using appropriate multiplex antibody bead kits purchased from Invitrogen (Invitrogen Corporation, Carlsbad, CA, USA). Data were analysed using StarStation version 2.0 software (Applied Cytometry Systems, Sheffield, UK). The lowest levels of detection were (pg/ml): TNF-α: 0.5, IL-1β: 1.0, IL-1Ra: 30.0, IL-6: 1.0, IL-10: 1.0, TNF-RI: 0.02, and TNF-RII: 0.02. Kit precisions were (CV%): TNF-α: 7.7, IL-1β: 4.4, IL-1Ra: 5.0, IL-6: 7.6, IL-10: 9.4, TNF-RI: 4.3, and TNF-RII: 7.9. The concentration of YKL-40 in plasma was determined by an ELISA assay (Kyidel, Santa Clara, CA, USA).

#### Oxidation markers

For the determination of the concentration of malondialdehyde (MDA), ascorbic acid (AA), dehydroascorbic acid (DHA) and total ascorbic acid (TAA) the blood samples were drawn into tubes containing heparin (LPS-free) and centrifuged at 3,100 rpm for 3 minutes and stored at −80° C until analysis. AA, DHA and TAA were stabilized with 10% meta-phosphoric acid containing 2 mM disodium EDTA before storage. Both MDA and AA were analysed using a high pressure liquid chromatograph method as described previously [18], [19].

### Statistics

The study population was set on empiric basis, because of lack of previous studies that will allow a power calculation. All data were tested for normality using the Kolmogorov-Smirnov test. Data that were not normally distributed were log-transformed and then became normally distributed. Measurement at different time points between the two study days were tested by two-way repeated measures of ANOVA. The two factors were time and study day (daytime or nighttime endotoxaemia). Between groups assessments at given time points data were not normally distributed, and Wilcoxon's rank test were used. The data are presented as means ± SE unless specified otherwise, and results with *P*-values<0.05 were considered statistically significant. SPSS version 20 software (SPSS, Chicago, Illinois, USA) was used for statistical analyses.

## Results

Twelve healthy young men were included in the study with a mean age of 23 years (range 19–31). All participants completed both study days with no missed samples. The wash-out period between the two study days were median 32 days (range 26–56).

### Day-night difference in the inflammatory response

**Figure 1:**
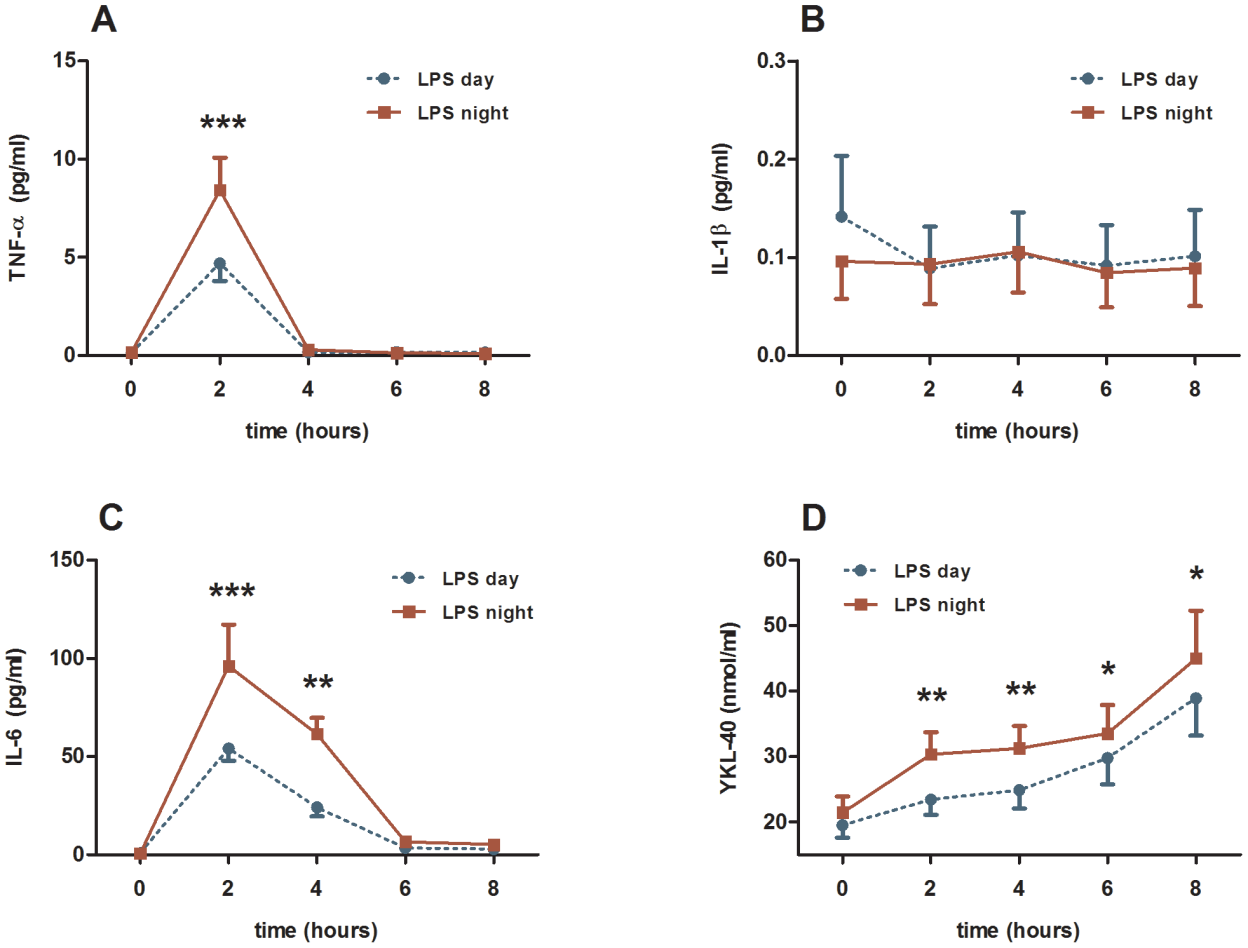
Plasma levels of three pro-inflammatory markers and YKL-40. The time point 0 indicates the administration of *E. coli* endotoxin (LPS). The endotoxaemia was induced at day time (blue curve) and night time (red curve). Results from the two-way ANOVA: (1) interaction term (time*day) were not significant for any of the markers. (2) between groups analyses were significant for IL-6 (*P*<0.0001) and YKL-40 (*P*<0.001). *) *P*-value<0.05 calculated by Wilcoxon-Rank test. **) *P*-value<0.01 calculated by Wilcoxon-Rank test. ***) *P*-value<0.001 calculated by Wilcoxon-Rank test.

**Figure 2:**
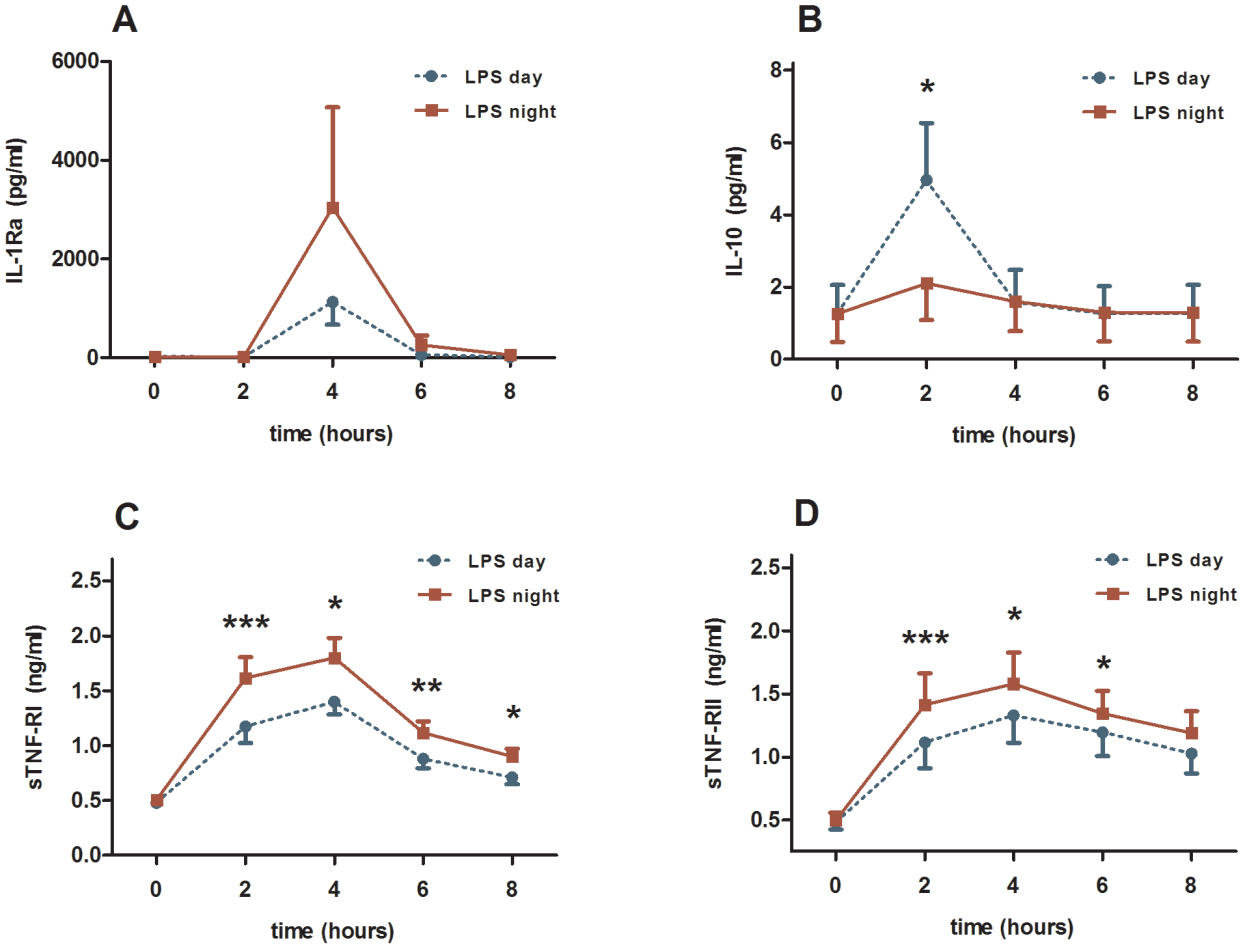
Plasma levels of four anti-inflammatory cytokines and soluble cytokine receptors. The time point 0 indicates the administration of LPS endotoxin 0.3/kg in 12 healthy men. The endotoxaemia was induced at day time (blue curve) and night time (red curve). Results from the two-way ANOVA: (1) interaction term (time*day) were significant for only IL-10 (*P*<0.001). (2) between groups analyses were significant for IL-1Ra (*P*<0.05), sTNF-RI (*P*<0.000000001) and sTNF-RII (*P*<0.000001). *) *P*-value<0.05 calculated by Wilcoxon-Rank test. **) *P*-value<0.01 calculated by Wilcoxon-Rank test. ***) *P*-value<0.001 calculated by Wilcoxon-Rank test.

Apart from IL-1beta, all pro- and anti-inflammatory cytokines, TNF-α, sTNF-RI and sTNF-RII, IL-1Ra, IL-6, IL-10 increased significantly 2 and/or 4 hours after induction of endotoxaemia compared with baseline levels (Figures 1 and 2; and table 1). Blood levels of the pro-inflammatory marker YKL-40 increased.

**Table 1:** Result of two way repeated measures of ANOVA.

Parameter	Between groups (day)	Interaction term (time * day)
IL-1β	ns	ns
TNF-α	ns	ns
IL-6	p<0.0001	ns
YKL-40	p<0.001	ns
IL-1Ra	p<0.05	ns
IL-10	ns	p<0.001
TNF-RI	p<0.000000001	ns
TNF-RII	p<0.000001	ns
MDA	p<0.05	p<0.05
AA	ns	ns
DHA	ns	ns

ns (not significant).

IL: ineterleukin.

TNF: tumor-necrosis factor.

MDA (malondialdehyde).

AA (ascorbic acid).

DHA (Dehydroascorbic acid).

The pro-inflammatory cytokine IL-6 (*P*<0.0001, two-way ANOVA, Figure 1C) and YKL-40 (*P*<0.001, Figure 1D), but not TNF-α showed significantly higher levels during night time endotoxaemia compared with daytime endotoxaemia. The anti-inflammatory cytokines/soluble cytokine receptors IL-1Ra (*P*<0.05, Figure 2A), sTNF-RI (*P*<0.0001, Figure 2C) and sTNF-RII (*P*<0.000001, Figure 2D) showed significantly higher levels during the night time compared with daytime, whereas IL-10, another anti-inflammatory cytokine, had significantly higher blood levels 2 hours after induction of endotoxaemia, but only with day time administration (*P*<0.05, Figure 2A). sTNF-RI and sTNF-RII showed significantly higher night-time levels at time points 2, 4, 6 and 8 hours (*P*<0.05, Figure 2C–D).

The inflammatory markers showed significantly different levels at the two study days at certain time points after the onset of endotoxaemia. YKL-40 was significantly higher during the night time at 2, 4, 6 and 8 hours post endotoxaemia (*P*<0.05, Figure 1D). TNF-α showed significantly higher levels during the night 2 hours after onset of endotoxaemia (*P*<0.01 Figure 1A). IL-6 levels were higher at night time 2, 4 and 6 hours post endotoxaemia (*P*<0.05, Figure 1C). IL-1Ra showed significantly higher levels at night at time point 4 hours (*P*<0.05, Figure 2B).

### Day-night difference in oxidative stress response

**Figure 3:**
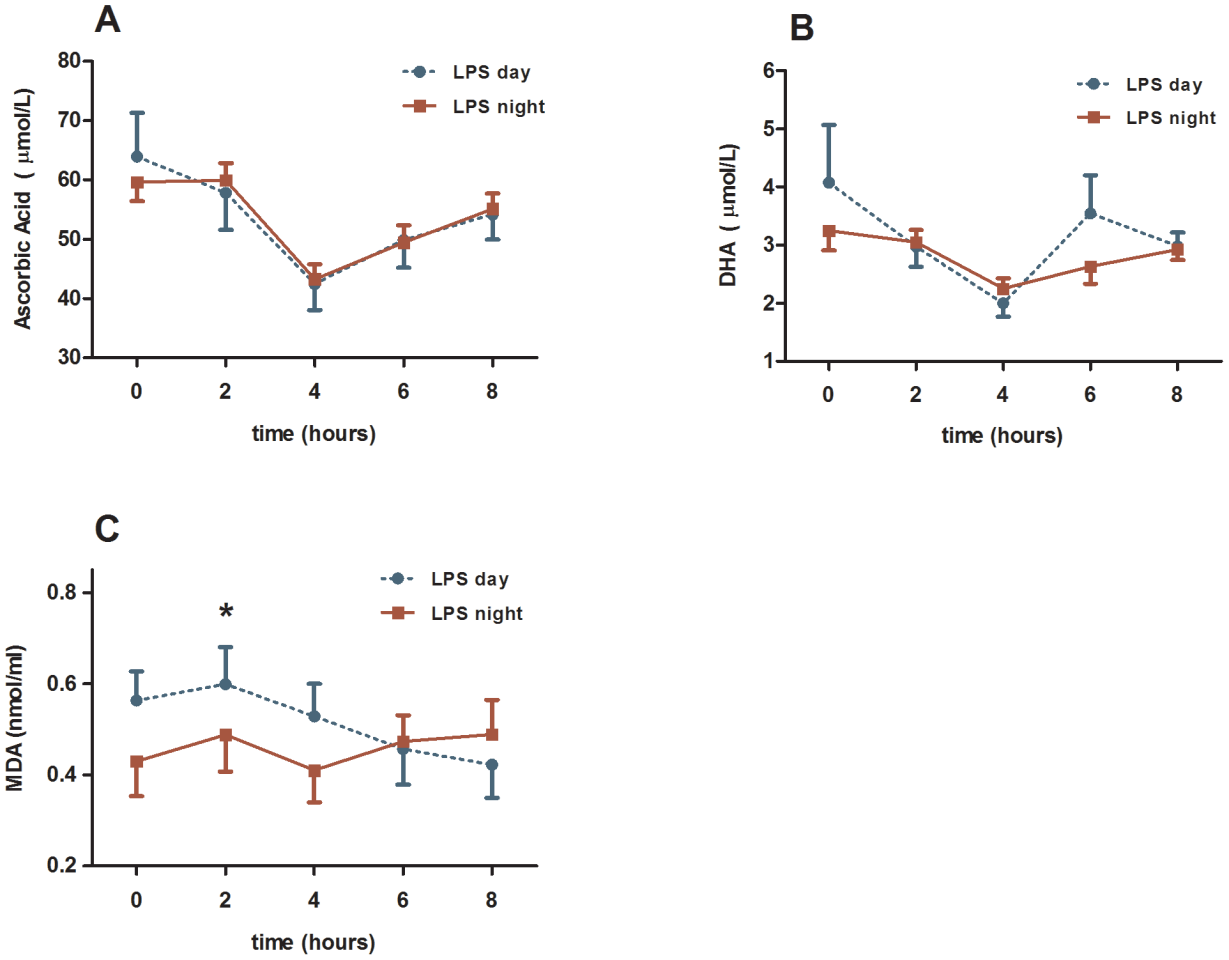
Plasma levels of the analysed oxidative markers. The time point 0 indicates the administration of LPS endotoxin 0.3/kg on 12 healthy men. The endotoxaemia was induced at day time (blue curve) and night time (red curve). Results from the two-way ANOVA: (1) interaction term (time*day) were significant for MDA (*P*<0.05). (2) between groups analyses were significant for MDA (*P*<0.05). *) *P*-value<0.05 calculated by Wilcoxon-Rank test.

The oxidative markers AA, DHA and MDA changed significantly after induction of endotoxaemia compared with baseline levels (*P*<0.05, Figure 3). The plasma levels of MDA, but not those of AA and DHA were significantly different at days versus nights. Thus, MDA was significantly higher during daytime (*P*<0.05, Figure 3C), both between groups and interaction term. At time point 2 hours after the onset of endotoxaemia MDA showed significantly higher values during daytime (*P*<0.05, Wilcoxon-Rank test). None of the statistical analyses showed significant differences between day and night for AA and DHA (Figure 3A–B).

### Day-night difference in vital parameters

**Figure 4:**
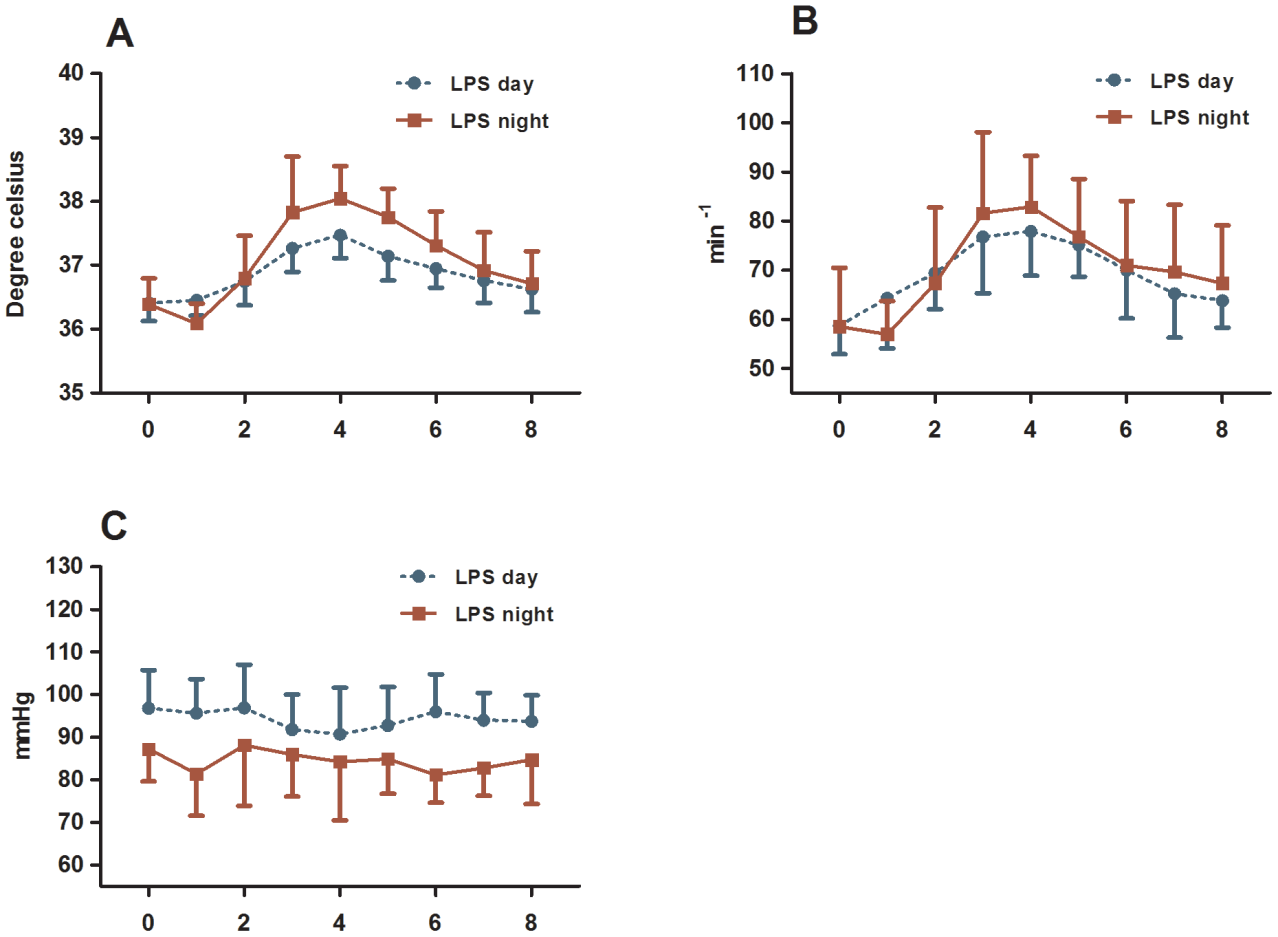
Body temperature (A), heart rate (B) and mean blood pressure (C) during endotoxaemia. The time point 0 indicates the time of LPS administration. Endotoxaemia was induced at day time (blue curve) and night time (red curve). Results from the two-way ANOVA: (1) interaction term (time*day) were not significant, (2) between groups analyses were significant for temperature (*P*<0.0001) and mean blood pressure (*P*<0.001).

Body temperature, heart rate but not mean blood pressure changed significantly after the administration of endotoxin (*P*<0.0001, Figure 4). A significantly higher body temperature (Figure 4A) and heart rate (Figure 4B) were seen at night time endotoxaemia compared to day time.

## Discussion

We have shown that the strong pro-inflammatory cytokines, TNF-α and IL-6, and YKL-40, showed a more pronounced response to LPS endotoxaemia during nighttime compared with daytime. The anti-inflammatory cytokine, IL-1Ra, and the soluble TNF-receptors, sTNF-RI and sTNF-RII, were also higher at night. The strong anti-inflammatory cytokine, IL-10, and the MDA as a parameter for oxidative damage was higher during day-time endotoxaemia. These results confirm that day-night variations in the acute phase response exist also at the cytokine level with an overall more pronounced inflammatory response during the night.

Sepsis is a systemic inflammatory response syndrome for instance due to an infection. Sepsis generally occurs in a heterogeneous group of patients, with or without prior surgical interventions, and frequently with multiple comorbidities. This complicates the interpretation of clinical investigations of sepsis pathophysiology, and it obstructs development of therapies for this potentially lethal condition. In humans, the most used and reliable model to study systemic inflammatory response is the human endotoxin model, in which LPS from *E. coli* or other Gram-negative bacteria is administered intravenously to healthy volunteers [14]. LPS endotoxin binds to Toll-like receptors on both blood leukocytes and endothelial cells initiating synthesis and secretion of pro-inflammatory and anti-inflammatory cytokines, as well as many other mediators [14].

TNF-α and IL-1beta are the archetype pro-inflammatory cytokines [14], [15]. These, and to a lesser extent IL-6, strongly promote inflammation. In contrast, anti-inflammatory cytokines, principally IL-10, but also IL-1Ra and the TNF-binding soluble TNF-receptors sTNF-RI and -II, inhibit inflammation [14]. The human endotoxaemia model results in many-fold increases in both the anti- and the pro-inflammatory cytokines [14].

YKL-40 is a lectin that is secreted by macrophages and neutrophils in patients with sepsis, bacterial pneumonia, meningitis, encephalitis, rheumatoid arthritis, inflammatory bowel diseases, and in patients with cancer [20], [21]. Blood YKL-40 levels are associated with the severity and fatal outcome of bacterial pneumonia and sepsis [21], [22], and endotoxaemia in human models induces elevated levels of YKL-40 [20].

Halberg et al. were the first to investigate the circadian variation in the inflammatory response due to endotoxaemia [8], demonstrating that it was higher during day compared with night in mice. This was later confirmed and extended in that endotoxaemia at day was associated with higher mortality in mice [9]. In our experimental model on rats, we demonstrated that the inflammatory response was higher at night compared to day, where both IL-6 and IL-10 were significantly higher at night compared to daytime endotoxaemia, indicating that this circadian rhythmicity are species dependant [10]. To our knowledge, the only previous study in humans was conducted by Pollmacher et al., who did not show any significant difference in the levels of TNF-α and IL-6 between day and night, but rather a higher levels of ACTH and cortisol at night [11]. The LPS endotoxin tolerance is present 2 weeks after an endotoxaemic response in healthy volunteers [23], [24] possibly explaining the lack of day/night difference in the study by Pollmächer et al.

Oxidative stress is defined as a disturbance in the balance between pro-oxidant factors and anti-oxidant factors in favour of the former leading to potential damage, also called oxidative damage. Measuring an increase in the oxidative stress-markers, such as reactive oxygen species and nitric oxide, does not necessarily indicate an increase in the oxidative damage. Endotoxaemia induces both increased inflammatory stress response and oxidative stress response [25], [26]. In the present study we included malondialdehyde (MDA), one of the breakdown products of lipid peroxidation and a marker of oxidative damage [27], [28].

To our knowledge, circadian variations in the levels of MDA in a human endotoxaemia model have not been previously described. The MDA levels were higher during day time compared with night time. Endotoxaemia induced by LPS has previously been shown to increase levels of MDA [29]. Daily variations in the levels of MDA are debatable, as there are studies reporting a circadian rhythm [30], [31] while other studies questions this circadian rhythm [32]. No day-night variation was seen in AA. In contrast to the inflammatory cytokine response, the changes in the oxidative stress response indicate an increase in the oxidative damage during day time compared with night time. This is contrary to the response in inflammation that was most pronounced during the night.

One of the explanations why the inflammatory response was more pronounced during the night might be due to the circadian rhythm of cortisol. The endogenous corticosteroid levels exhibits a circadian rhythm with a peak during day and lowest levels during the night [9]. The anti-inflammatory effect of cortisol is thus lacking during the night, which might result in a more severe inflammatory response at night time [11], [33]. We did not measure the levels of corticosteroids in our study.

Another explanation of the diurnal variation in endotoxic response is the interaction between clock genes and LPS. Clock genes have been identified in several tissues and peripheral blood cells, including macrophages. In humans, the only study examining the effect of LPS on clock genes was made by Haimovich *et al.* [34]. They found that LPS suppressed as much as 90% of the gene expression of PER1, PER2 and other clock genes in human peripheral blood leukocytes (PBL) for at least 17 hours. Furthermore, they demonstrated that melatonin secretion was not impaired by LPS, indicating that the circadian rhythm between the clock genes in the hypothalamus and clocks genes in PBL were disrupted by endotoxin [34]. However, others have found that melatonin secretion from the pineal gland is inhibited and reduced by LPS and TNF-α [35]

Circadian variation in the inflammatory response to endotoxin may have serious clinical implications. For example, TNF-α administration to mice showed a nine-fold increase in the lethality through the day [36]. The lethality was highest when TNF-α was administered just before awakening and the survival were highest when the administration was in the second half of the light period. Mortality was also higher when endotoxaemia was initiated in mice at day time compared with night time [9]. One explanation why these data do not correlate to our findings could be, that mice are physically active at the dark period of the day, while humans are physically active at the light period of the day. While humans are diurnally active, mice are active both day and night having an opposite rhythm of glucocorticoid compared to humans. The interaction between immune system and pineal gland was investigated in a recent study, where patients undergoing hysterectomy showed an inverse relation between plasma levels of melatonin and TNF-α [37]. Higher postoperative levels of TNF-α and lower levels of cortisol were accompanied by lower levels of melatonin.

In our study, we included only male subjects. Several aspects lead us to include solely men in this trial: the influences of the menstrual cycle on the levels of and the rhythmicity of melatonin and cortisol [38]; menstrual cycle is associated with fluctuations in melatonin production [39]; endotoxin inhibits pulsatile LH and GnRH [40]; endotoxin stimulated mononuclear cells from men produce more TNF-α, IL-10 and higher expression of TLR4 compared to women [41]; and finally the amount of released TNF-α and IL-6 from LPS-induced monocytes varies during the menstrual cycle, highest in the follicular phase and lowest in the luteal phase [42], [43].

Our trial was an experimental study with endotoxaemia, which imitates the initial phase of sepsis, and therefore cannot be used to study sepsis pathology beyond the acute phase response. We applied a dosage of LPS of 0.3 ng/kg b.w. in this trial, which induces a moderate acute phase response as seen in the levels of cytokines. Higher doses of endotoxin can initiate responses that may be resistant to day-night differences. Sepsis can also be initiated by different pathogen-associated molecular patterns other than LPS, i.e. peptidoglycan, which may initiate sepsis through other cellular pathways resulting in a different acute phase response. Body temperature measurement was assessed by ear thermometer which is influenced by several confounders. In this cross over study, the size of the study population was based on empiric estimation. A post-hoc power calculation based on mean and standard deviation for TNF-α on t = 2, gives a 61% post-hoc power. Normally, the statistical power is set to be 80%. According to this post-hoc calculation, we need more than 18 subjects in each group to have an 80% statistical power.

In conclusion, we have shown that endotoxin-induced inflammatory and oxidative damage exhibit day-night difference, where the inflammatory response is more pronounced at night and the oxidative damage is more pronounced at daytime. Investigations of the circadian variations in inflammatory responses in humans appear warranted, also because of the prophylactic and therapeutic implications. Finally, because melatonin has been shown to have a potent anti-inflammatory and anti-oxidative effect in experimental studies, it could be interesting to investigate whether melatonin administration at night may reduce the inflammatory and oxidative damage associated with sepsis.
